# Living Donor Liver Transplantation in Patients with or Without Acute-on-Chronic Liver Failure: A Single Center Experience

**DOI:** 10.3390/jcm15083007

**Published:** 2026-04-15

**Authors:** Bandar Aljudaibi, Lama Alshehri, Bedour Almudaiheem, Samra Mirza, Ahmad Mirza, Aiko Danish, Mir Hakam Qazi, Mohammed Shoaib, Yousef Hamed, Massimo Malago, Dimitri Raptis, Saleh Alabbad, Fuat Saner, Saad Alghamdi, Abdullah Alfhaid, Ehab Abufarhaneh, Saleh Alqahtani, Dieter Broering, Khalid Bzeizi

**Affiliations:** 1 Organ Transplant Centre of Excellence, King Faisal Specialists and Research Centre, Riyadh 11211, Saudi Arabia; lmalshehri@kfshrc.edu.sa (L.A.); bmudaiheem@kfshrc.edu.sa (B.A.); mmalago@kfshrc.edu.sa (M.M.); draptis@kfshrc.edu.sa (D.R.); salehalabbad@kfshrc.edu.sa (S.A.); fsaner@kfshrc.edu.sa (F.S.); aalfhaid@kfshrc.edu.sa (A.A.); ehab@kfshrc.edu.sa (E.A.); salalqahtani@kfshrc.edu.sa (S.A.); dbroering@kfshrc.edu.sa (D.B.); kbzeizi@kfshrc.edu.sa (K.B.); 2 College of Medicine, Alfaisal University, Riyadh 11533, Saudi Arabia; smirza@alfaisal.edu.sa (S.M.); amirza@alfaisal.edu.sa (A.M.); adanish@alfaisal.edu.sa (A.D.); dtps.med@gmail.com (M.H.Q.); milyas@alfaisal.edu.sa (M.S.); yhamed@alfaisal.edu.sa (Y.H.)

**Keywords:** acute-on-chronic liver failure, living donor liver transplantation, chronic liver disease, MELD-Na score, perioperative complications, long-term survival, ACLF grading, chronic kidney disease, graft survival

## Abstract

**Background/Objectives:** Acute-on-chronic liver failure (ACLF) is a severe syndrome in chronic liver disease (CLD) patients, characterized by multi-organ failure and high mortality. Living donor liver transplantation (LDLT) is vital in donor-scarce areas. This study compares baseline characteristics, perioperative complications, and long-term survival between ACLF and non-ACLF patients, emphasizing etiology, ACLF grading, and graft factors. **Methods:** Data from a prospective registry of 591 adult LDLT recipients (2019–2023) were analyzed. ACLF was defined by EASL-CLIF (multi-organ failure, grades 1–3) and APASL (jaundice/coagulopathy with complications) criteria, evaluated at initial assessment and within 24 h pre-LDLT. **Results:** ACLF patients (*n* = 101, 17.1%) showed higher MELD-Na (27 vs. 20, *p* < 0.001), bilirubin (6.84 vs. 1.75 mg/dL, *p* < 0.001), creatinine (108 vs. 70.5 μmol/L, *p* < 0.001), metabolic/genetic etiologies (9.9% vs. 2.8%, *p* = 0.001), and chronic kidney disease (23.7% vs. 8.1%, *p* < 0.001), and lower HCC rates (11.8% vs. 29.6%, *p* < 0.001). GRWR was marginally lower in ACLF patients (0.59 vs. 0.66, *p* = 0.10). The ACLF group had elevated infection (27.7% vs. 10.4%, *p* < 0.001), bleeding (14.9% vs. 6.3%, *p* = 0.004), and biliary complications (15.8% vs. 7.8%, *p* = 0.010), with longer ICU (5 vs. 3 days, *p* < 0.001) and hospital stays (33.66 vs. 20.7 days, *p* = 0.036). Five-year overall survival was reduced in ACLF patients (log-rank *p* = 0.001), worsening with grade (EASL-CLIF grade 3: 55% vs. 81% for no ACLF, *p* = 0.002). Graft survival was also lower (75% vs. 85%, *p* = 0.02). Multivariable analysis identified chronic kidney disease as an independent mortality predictor (HR 2.09, 95% CI 1.11–3.95, *p* = 0.023). **Conclusions:** LDLT for ACLF involves higher perioperative risks and poorer long-term survival than non-ACLF patients, with outcomes deteriorating by ACLF grade. Chronic kidney disease independently predicts mortality. Timely LDLT is essential in donor-limited regions.

## 1. Introduction

Acute-on-chronic liver failure (ACLF) represents one of the most critical syndromes in hepatology, characterized by acute decompensation in patients with underlying chronic liver disease (CLD), systemic inflammation, immune dysfunction, multi-organ failure, and very high short-term mortality rates [1,2]. Globally, ACLF affects an estimated 30–40% of hospitalized cirrhosis patients, with a 28-day mortality ranging from 18% in grade 1 to nearly 90% in grade 3 without transplantation [3,4].

Two major classification systems dominate current practice: the European Association for the Study of the Liver–Chronic Liver Failure (EASL-CLIF) consortium definition and the Asian Pacific Association for the Study of the Liver (APASL) criteria [3,4]. The EASL-CLIF system, based on the CLIF-SOFA score, emphasizes multi-organ dysfunction, grading ACLF into three stages depending on the number and severity of organ failures [3]. It captures systemic inflammatory burden and predicts mortality more accurately in Western populations, where alcohol and infections predominate [3,5,6]. In contrast, the APASL criteria are more hepatocentric, focusing on acute hepatic insults manifesting as jaundice (bilirubin ≥ 5 mg/dL) and coagulopathy (INR ≥ 1.5), with rapid onset of ascites or encephalopathy within 4 weeks [4]. This reflects the viral hepatitis-driven epidemiology of ACLF in Asia. While EASL-CLIF may better stratify multi-organ dysfunction, APASL remains valuable in viral-dominant regions, and combined frameworks are being proposed for harmonization [7].

The prognosis of ACLF without liver transplantation is very poor, particularly in advanced grades, where mortality may exceed 70% within three months [3,6]. Supportive care, antivirals for HBV, and intensive organ support can stabilize subsets, but liver transplantation (LT) remains as the definitive therapy. Both deceased donor liver transplantation (DDLT) and living donor liver transplantation (LDLT) have been applied in ACLF, but important differences exist. DDLT remains the gold standard in Western countries, but waitlist mortality is exceedingly high in ACLF due to organ scarcity, hemodynamic instability, and rapid deterioration [8,9]. LDLT, more prevalent in Asia and increasingly in the Middle East, enables timely transplantation, bypassing prolonged waiting periods, and has demonstrated survival benefits for ACLF patients [7,10,11]. However, LDLT carries unique risks, including small-for-size syndrome when graft-to-recipient weight ratio (GRWR) is below 0.6, potentially compounding the morbidity of ACLF [12,13].

Existing literature provides heterogeneous findings on ACLF outcomes after LT. Some series suggest acceptable post-transplant survival comparable to non-ACLF recipients [7,14], while others highlight elevated perioperative risks and reduced short-term outcomes in multi-organ failure [10,15]. Regional differences in etiology further complicate interpretation. In East Asia, HBV reactivation is predominant [16], in Europe, alcohol-related liver disease dominates [5], and in the Middle East, metabolic dysfunction-associated steatotic liver disease (MASLD) is rising rapidly [17]. These distinctions raise the need for geographically oriented and structured data.

In this study, we aimed to evaluate long-term outcomes of LDLT in ACLF versus non-ACLF patients at a high-volume tertiary transplant center. By integrating EASL-CLIF and APASL classifications alongside graft factors, this study addresses critical knowledge gaps and informs strategies for ACLF management in donor-scarce regions.

## 2. Materials and Methods

### 2.1. Study Design and Population

This retrospective cohort study with data derived from a local prospectively maintained registry included 591 consecutive adult patients (>18 years) undergoing LDLT at King Faisal Specialist Hospital and Research Centre, Riyadh, from January 2019 to August 2023, with follow-up extended to April 2025. Exclusions included re-transplants, multi-organ transplants, and records with >5% missing data.

ACLF was diagnosed using EASL-CLIF (acute decompensation with ≥1 organ failure, graded 1–3 via CLIF-SOFA) and APASL (jaundice [bilirubin ≥ 5 mg/dL], INR ≥ 1.5, with ascites/encephalopathy within 4 weeks) criteria, assessed at admission and 24 h pre-transplant. Patients were classified as ACLF when they fulfilled APASL criteria for acute hepatic decompensation and demonstrated organ failure according to the EASL-CLIF system.

Chronic kidney disease was defined according to KDIGO criteria as an estimated glomerular filtration rate < 60 mL/min/1.73 m^2^ for more than 3 months.

ACLF grade immediately prior to transplantation was used for outcome analyses.

### 2.2. Data Collection

Data were extracted from the Centre’s transplant registry and electronic health records, comprising recipient demographics (age, gender, BMI); comorbidities (diabetes, hypertension, hepatocellular carcinoma [HCC]); laboratory parameters (MELD-Na, INR, bilirubin, creatinine); clinical signs (jaundice, ascites, gastrointestinal bleeding); and transplant indications (metabolic/genetic, viral, MASLD, cryptogenic, immune-mediated).

#### 2.2.1. Donor Characteristics

All liver grafts were obtained from living donors who underwent a standardized evaluation protocol including medical, surgical, and psychosocial assessment.

Donor variables included age, sex, body mass index, graft weight, and graft-to-recipient weight ratio (GRWR). Graft types consisted primarily of right-lobe grafts, with left-lobe grafts used selectively when appropriate.

All transplantations were performed with ABO-compatible donor–recipient pairs.

#### 2.2.2. Surgical Procedure:

Recipient hepatectomy and graft implantation were performed using standardized surgical techniques at our center.

Warm ischemia time was defined as the interval between removal of the graft from cold preservation and completion of portal vein reperfusion. Due to the nature of LDLT and immediate implantation, this period is typically short.

Intraoperative variables included operative duration, estimated blood loss, and transfusion requirements. Operative duration for both donor hepatectomy and recipient transplantation along with blood loss and transfusions was recorded.

Further data were Comprehensive Complication Index [CCI], ICU/hospital stay, readmissions, re-transplantation, and 5-year patient/graft survival. Follow-up was censored at death, graft loss, or last contact.

#### 2.2.3. Immunosuppression Protocol

Post-transplant immunosuppression consisted of a standard triple-drug regimen including tacrolimus, mycophenolate mofetil, and corticosteroids. Corticosteroids were gradually tapered over the first three months following transplantation, unless required for a longer duration.

In selected patients, particularly those with renal dysfunction, basiliximab induction therapy was used to allow delayed initiation or reduced exposure to calcineurin inhibitors.

Artificial intelligence was used for linguistic polishing and the final text was reviewed and approved by the authors.

### 2.3. Statistical Analysis

Data analysis was conducted using STATA (Release 18.0) and R (version 4.5.2) [18,19]. The Mann–Whitney U test was used to compare continuous variables, represented by medians and interquartile ranges (IQRs). Categorical variables, expressed as counts and percentages, were analyzed using the Chi-square test. Kaplan–Meier survival and log-rank tests were used to evaluate survival, categorized by ACLF status and APASL grade. To identify survival predictors, multivariable Cox regression analysis was performed, adjusting for ACLF and APASL grade. A significance level of 0.05 was applied, indicating statistical significance with a 5% margin of error and a 95% confidence level for all analyses.

### 2.4. Ethical Considerations

The study was approved by the Institutional Review Board of King Faisal Specialist Hospital with a consent waiver due to its retrospective design. The IRB tracking number is RAC 2251200.

## 3. Results

Of 591 adult LDLT recipients, 101 (17.1%) fulfilled ACLF criteria and 490 (82.9%) did not. Baseline recipient characteristics are shown in Table 1. Patients with ACLF had significantly higher disease severity at transplant, with a median MELD-Na score of 27 (IQR 23–32) vs. 20 (IQR 16–22; *p* < 0.001), total bilirubin 6.84 mg/dL (IQR 2.1–23.2) vs. 1.75 mg/dL (IQR 0.9–3.3; *p* < 0.001), and serum creatinine 108 μmol/L (IQR 64–205) vs. 70.5 μmol/L (IQR 56–89; *p* < 0.001). Chronic kidney disease was more frequent in the ACLF group (23.7% vs. 8.1%; *p* < 0.001), and among ACLF grade 1 patients, renal dysfunction represented the most common extra-hepatic organ failure. Hemodialysis prior to transplantation was required in 24 patients, including 15 in the ACLF group and nine in the non-ACLF group.

HCC was less prevalent among ACLF recipients (11.8% vs. 29.6%; *p* < 0.001). Metabolic/genetic etiology was over-represented in ACLF (9.9% vs. 2.8%; *p* = 0.001). Age and sex distribution were similar between groups.

Donor characteristics (Table 1) were comparable, although graft-to-recipient weight ratio (GRWR) trended lower in ACLF recipients (median 0.59 vs. 0.66; *p* = 0.10).

### 3.1. Perioperative Outcomes

Post-operative complications and intraoperative data are summarized in Table 2. ACLF patients experienced significantly higher rates of infection (27.7% vs. 10.4%; *p* < 0.001), major bleeding (8.9% vs. 4.4%; *p* = 0.007), and biliary complications (15.8% vs. 7.8%; *p* = 0.010). Severe complications (Clavien-Dindo ≥ 3) occurred in 35.6% of ACLF vs. 20% of non-ACLF patients (*p* = 0.001). The Comprehensive Complication Index was markedly elevated in ACLF patients (median 20.9 vs. 0; *p* = 0.002). Resource utilization was greater in the ACLF cohort: median ICU stay was 5 days (IQR 3–10) vs. 3 days (IQR 3–5; *p* < 0.001) and hospital stay 26 days (IQR 18–42) vs. 20 days (IQR 5–28; *p* = 0.0001). Ninety-day readmission rates were 33.6% vs. 23.6% (*p* = 0.036). Re-transplantation rates did not differ significantly.

Intra-operative data demonstrated greater blood loss in ACLF patients (median 2800 mL vs. 1200 mL; *p* < 0.001) and more blood transfusions (1750 mL vs. 1000 mL; *p* < 0.001).

### 3.2. Survival Outcomes

**Figure 1:**
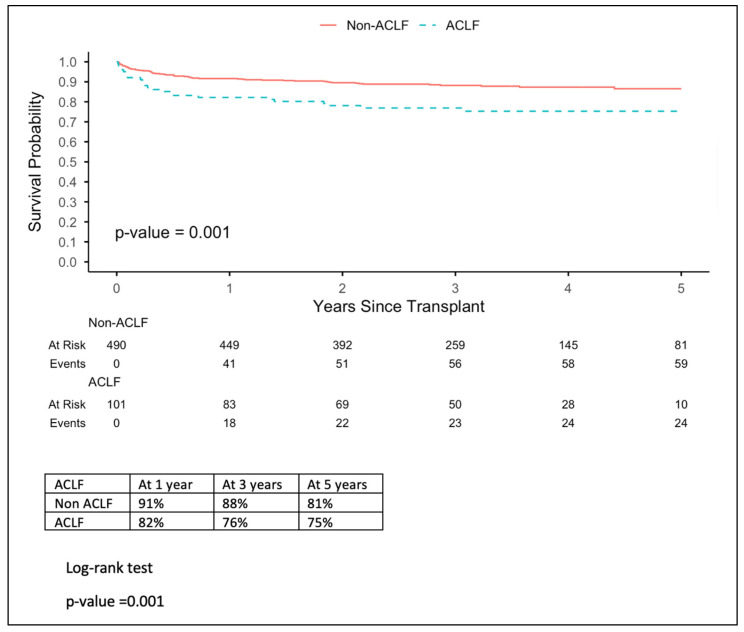
Overall patient survival (by ACLF). ACLF: Acute-on-chronic liver failure.

**Figure 2:**
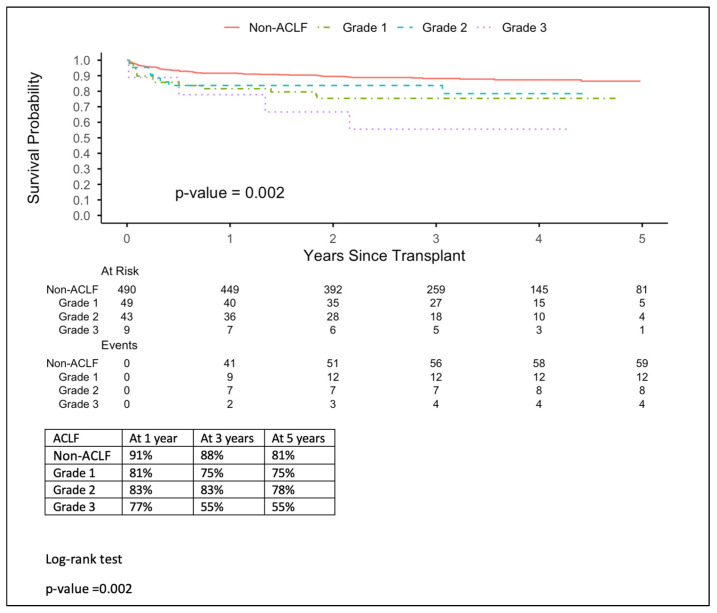
Acute-on-chronic liver failure (ACLF) grade and patient survival.

**Figure 3:**
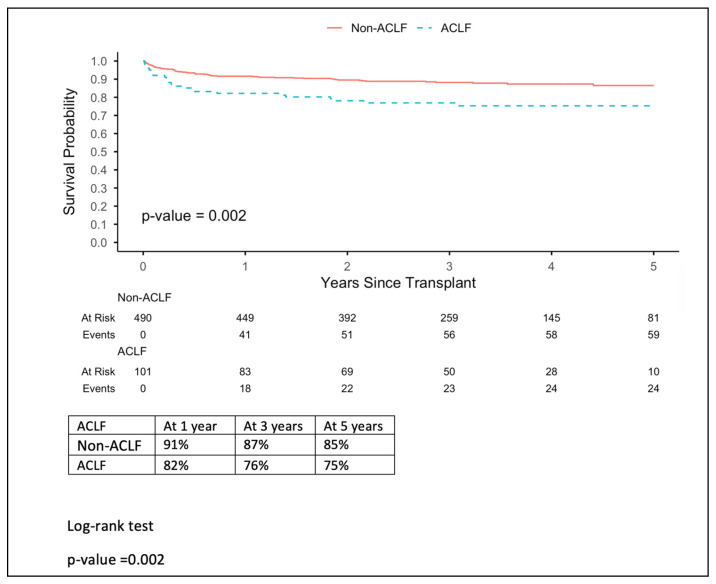
Overall graft survival (by acute-on-chronic liver failure [ACLF]).

Kaplan–Meier estimates of 5-year patient survival differed significantly by ACLF status, *p*-value = 0.001. Non-ACLF patients had superior survival, with 81% at the end of the survival period, compared with 75% for ACLF patients (Figure 1). Survival declined steeply with increasing ACLF severity. Grades 1, 2, and 3 represent survival percentages 75%, 78%, and 55%, respectively, which show that grade 3 represents the lowest survival rate by outcome (Figure 2). On the other hand, the APASL grading showed 78% survival for grade 2, which is the lowest value in comparison to grades 1 and 3 (89% and 82%, respectively). Moreover, grades 2 and 3 start with similar percentages until the third year of follow-up; grade 2 continues to drop, whereas grades 3 and 1 remain steady. Graft survival mirrored patient survival overall; 5-year graft survival is lower in ACLF patients (75% vs. 85%), with a *p*-value of 0.02 (Figure 3). In addition, APASL grade showed no difference in graft survival from patient survival, with a *p*-value of 0. 02.

Multivariable Cox regression identified kidney disease as an independent predictor of mortality (HR 2.09, 95% CI 1.11–3.95; *p* = 0.023). After adjustment for age, MELD-Na, GRWR, and etiology, neither ACLF nor GRWR <0.6 retained independent significance (Table 3).

Dynamic changes in ACLF grade between initial evaluation and 24 h pre-transplant were evaluated. Most patients in grades 0 and 1 remained stable, whereas a substantial proportion of those initially graded 2 and 3 improved to lower grades by the time of transplantation; a minority of lower grades progressed.

## 4. Discussion

This large, single-center analysis provides important insights into the role of LDLT in patients with ACLF. Our findings highlight the duality of outcomes: while perioperative morbidity and hospital resource utilization are significantly higher in ACLF compared to non-ACLF patients, long-term survival is reduced, with clear declines in advanced ACLF grades. Chronic kidney disease emerged as the only independent predictor of mortality in multivariable analysis, underscoring its critical role in post-LDLT prognosis.

Comparison with prior studies confirms that patients with ACLF present higher severity of illness at transplant, reflected in elevated MELD-Na, bilirubin, and creatinine, and greater prevalence of renal dysfunction [6,10,14]. The higher proportion of metabolic etiologies in our Middle Eastern cohort emphasizes the epidemiologic transition from viral hepatitis towards MASLD-driven ACLF [16]. This differs from Asian data, where HBV remains the leading etiology [17], and from Western cohorts, where alcohol and sepsis predominate [5,11].

One of the most debated issues is whether outcomes of ACLF after transplantation differ substantially between LDLT and DDLT. Reports from Asia, where LDLT predominates, demonstrate 1- and 5-year survival rates of approximately 70–85% in advanced ACLF, with 5-year graft survival reaching 70.5% in high-MELD ACLF cohorts (including grade 3) [7,10,14,20]. For instance, in a large Korean LDLT series, 5-year survival for ACLF grade 3 was 71%, reflecting the benefits of timely grafting despite elevated risks [14]. By contrast, DDLT outcomes in Europe and the US are constrained by donor availability and prolonged waiting times, with many ACLF patients dying before transplant [21]. Nevertheless, once transplanted, short- and long-term survival after DDLT can approach that of non-ACLF recipients, with 1-year survival in ACLF grade 3 reaching 81.8% in multi-organ failure cohorts, particularly in carefully selected patients [10,22]. Our study confirms that LDLT allows timely transplantation in critically ill ACLF patients, mitigating waitlist mortality, but long-term survival remains inferior to non-ACLF recipients, particularly in advanced grades. The notably poorer 5-year survival in EASL-CLIF grade 3 ACLF (55% vs. 86% in non-ACLF) aligns with broader literature reporting 1-year post-LT survival as low as 68–82% in this subgroup, driven by persistent multi-organ dysfunction [10,14,23]. In our cohort, this disparity is likely multifactorial, with pre-existing kidney disease emerging as a dominant independent risk factor. Renal impairment in ACLF grade 3 often reflects a combination of hepatorenal syndrome, acute kidney injury from sepsis or hypoperfusion, and underlying chronic kidney disease, leading to prolonged post-LT dialysis dependence in up to 30–50% of severe cases across studies [24,25]. The paucity of combined liver–kidney transplants in donor-scarce regions like the Middle East exacerbates this, as sequential kidney allocation post-LT is limited, contributing to late infections, cardiovascular events, and cumulative morbidity [26]. Future analyses in our registry will quantify dialysis duration and renal recovery rates to refine these insights, but current evidence supports aggressive pre- and post-transplant renal support, including early dialysis initiation and multidisciplinary care, to mitigate long-term risks in grade 3 patients.

The dynamic nature of ACLF severity highlights the importance of repeated assessments. Patients with initially severe grades (2 and 3) who improved following transplant had better outcomes, suggesting that stabilizing ACLF prior to surgery may improve survival. Conversely, progression to higher grades underscores the need for urgent transplantation in deteriorating patients.

Although GRWR trended lower in ACLF and has been implicated in small-for-size syndrome, it did not emerge as an independent predictor in multivariable analysis. This likely reflects refined surgical and perioperative practices, including careful graft selection and portal flow modulation. Our study also highlights the resource-intensive nature of ACLF transplantation. Infections, bleeding, biliary complications, and longer ICU/hospital stays were all significantly more frequent, consistent with prior LDLT and DDLT series [27,28]. These complications reflect the immunologic and physiologic instability of ACLF, reinforcing the importance of perioperative critical care, multidisciplinary coordination, and infection prophylaxis.

Our analysis, consistent with most transplant series, includes only patients who successfully underwent LDLT and does not capture ACLF candidates who died while awaiting a donor, experienced clinical deterioration, or were deemed unsuitable for surgery. Consequently, the reported 55% five-year survival in EASL-CLIF grade 3 reflects outcomes in a highly select transplant-eligible subgroup and likely overestimates survival at the population level, representing an important limitation of this study. In addition, subgroup analyses, particularly for EASL-CLIF grade 3, were constrained by small sample sizes. The number of patients with ACLF grade 3 was relatively small in our cohort, which limited the statistical power of subgroup analyses.

A key strength of this study is its assessment of EASL-CLIF scores, both at the time of initial liver transplant evaluation and within 24 h of transplantation, enabling a dynamic evaluation of ACLF severity and its impact on outcomes. The updated analysis further clarifies the prognostic value of EASL-CLIF for patient and graft survival over APASL. The limitations of our study are primarily its retrospective nature and being confined to one center.

Ethical considerations also arise in LDLT for ACLF. Living donors are exposed to operative risk, and their grafts for recipients with advanced ACLF and high perioperative mortality risk must be carefully justified. Nonetheless, the demonstrated survival benefit supports LDLT as a justifiable intervention in well-selected candidates within donor-scarce regions.

## 5. Conclusions

LDLT provides a life-saving option for ACLF patients, offering long-term survival despite elevated perioperative risks and reduced survival compared to non-ACLF patients. EASL-CLIF grading and kidney disease are critical prognostic factors. In donor-scarce regions, LDLT should be prioritized for ACLF candidates to reduce waitlist mortality. Future multicenter prospective studies are needed to refine risk stratification, optimize graft selection, and evaluate interventions to mitigate perioperative morbidity.

## Figures and Tables

**Table 1:** Patient and donor characteristics.

Patient Characteristics	*N* = 591	No ACLF N = 490	ACLF N = 101	*p*-Value
Age (median, years)	58	58	58	0.23
Male (n, %)	366	298 (60.8%)	68 (67.3%)	0.22
Body mass index	26.2	26.15	26.3	0.97
MELD-Na	22	20	27	<0.001
INR	1.6	1.5	2.1	<0.001
Total bilirubin (mg/dL)	2	1.75	6.84	<0.001
Creatinine (mmol/L)	72	70.5	108	<0.001
Hepatocellular carcinoma (n, %)	157	145 (29.6%)	12 (11.8%)	<0.001
Chronic kidney disease (n, %)	64	40 (8.1%)	24 (23.7%)	<0.001
Lung disease (n, %)	51 (8.63)	41 (8.3%)	10 (9.9%)	0.61
Diabetes (n, %)	162 (27.4%)	132 (26.9%)	30 (29.7%)	0.57
Heart Disease (n, %)	45 (7.6%)	35 (7.1%)	10 (9.9%)	0.34
Hypertension (n, %)	385 (65.1%)	319 (65.1%)	66 (65.3%)	0.96
Ascites (n, %)	223 (37.7%)	185 (37.7%)	38 (37.6%)	0.98
Gastrointestinal bleeding (n, %)	377 (63.7%)	309 (63.0%)	68 (67.3%)	0.41
Indication for transplant (n, %)				
Metabolic/genetic	24 (4.1%)	14 (2.8%)	10 (9.9%)	<0.001
Viral	197 (33.3%)	162 (33.1%)	35 (34.6%)	0.757
MASLD	169 (28.6%)	141 (28.8%)	28 (27.7%)	0.83
Cryptogenic cirrhosis	46 (7.7%)	40 (8.16)	6 (5.9%)	0.448
Immune	105 (17.7%)	87 (17.76)	18 (17.8)	0.987
**Donor Characteristics**				
Age	28	28	29	0.27
Male (n, %)	495 (70.8%)	404 (70.6%)	91 (71.6%)	0.81
Body mass index	24.6	24.5	25	0.69
GRWR	0.65	0.66	0.59	0.10
Graft weight (grams)	669	667	676	0.28

MASLD: metabolic dysfunction-associated steatotic liver disease, GRWR: graft recipient weight ratio.

**Table 2:** Surgical recipient outcome and intra-operative data.

Surgical Recipient Outcome	N = 591	No ACLF N = 490	ACLF N = 101	*p*-Value
Acute kidney injury (n, %)	22	17 (3.4%)	5 (4.9%)	0.474
Infection (n, %)	79	51 (10.4%)	28 (27.7%)	<0.001
Bleeding (n, %)	31	22 (4.4%)	9 (8.9%)	0.070
Bile leak (n, %)	27	17 (3.4%)	10 (9.9%)	0.005
Biliary complications (n, %)	54	38 (7.8%)	16 (15.8%)	0.01
Portal vein thrombosis (n, %)	11	11 (2.2%)	0	0.129
Hepatic artery thrombosis/reconstruction (n, %)	31	25 (5.1%)	6 (5.94%)	0.731
Hepatic vein complication (n, %)	1	1 (0.2%)	0	0.650
Deep vein thrombosis (n, %)	5	5 (1.02%)	0	0.308
Pulmonary embolism (n, %)	1	1	0	0.650
Length of intensive care unit stay (days)	4 (3)	3 (2)	5 (7)	<0.001
Length of hospital stay (days)	20 (15)	20 (13)	26 (24)	0.0001
Readmission to the intensive care unit (n, %)	45 (8%)	36 (7.7%)	9 (9.3%)	0.589
Readmission rate within 90 days	150 (25.3%)	116 (23.6%)	34 (33.6%)	0.036
Re-transplant	10 (1.69%)	9 (1.8%)	1 (0.9%)	0.548
Clavien-Dindo ≥ 3	134 (22.67)	98 (20%)	36 (35.64%)	0.001
Comprehensive Complication Index	0 (26.2)	0 (20.9)	20.9 (33.8)	0.002
Intra-operative data				
Duration of recipient surgery (hours)	6	6	6.7	
Blood loss (mL)	1500 (2200)	1200 (1500)	2800 (3000)	<0.001
Blood transfusion (mL)	1000 (2000)	1000 (1750)	1750 (2000)	<0.001
Cold ischemia time (minutes)	86	85	89	0.254
Warm ischemia time (minutes)	7	7	7	0.467

ACLF: Acute-on-chronic liver failure.

**Table 3:** Outcome predictors.

Predictor Variable	Haz. Ratio	*p*-Value	95% Confidence Interval
ACLF	1.663	0.162	0.815–3.393
Viral	0.637	0.175	0.332–1.221
NASH	0.872	0.661	0.473–1.605
MELDNa	1.025	0.341	0.972–1.083
INR (24 h pre-transplant)	0.947	0.619	0.765–1.172
HCC	0. 508	0.056	0.254–1.016
Recipient age at transplant	1.015	0.158	0.993–1.038
Total bilirubin (mg/dL)	0.986	0.519	0.947–1.027
Chronic kidney disease	2.092	0.023	1.108–3.95
Diabetes	1.111	0.696	0.641–1.948
Lactate 24 h Pre Tx	1.038	0.640	0.888–1.213
Dialysis 24 h Pre Tx	1.111	0.880	0.279–4.42
Vasopressors 24 h Pre Tx	0.753	0.436	0.37–1.534
SpO2 24 h Pre Tx	0.944	0.338	0.839–1.061

ACLF: Acute-on-chronic liver failure, HCC: hepatocellular carcinoma, MELDNa: model for end-stage liver disease–sodium, INR: international normalized ratio.

## Data Availability

Data is available upon request.

## Abbreviations

The following abbreviations are used in this manuscript:

ACLF	Acute-on-Chronic Liver Failure
CLD	Chronic Liver Disease
LDLT	Living Donor Liver Transplantation
EASL-CLIF	European Association for the Study of the Liver–Chronic Liver Failure
APASL	Asian Pacific Association for the Study of the Liver
MELD-Na	Model for End-Stage Liver Disease–Sodium
CKD	Chronic Kidney Disease
HCC	Hepatocellular Carcinoma
GRWR	Graft-to-Recipient Weight Ratio
ICU	Intensive Care Unit
HR	Hazard Ratio
CI	Confidence Interval
CLIF-SOFA	Chronic Liver Failure–Sequential Organ Failure Assessment
INR	International Normalized Ratio
HBV	Hepatitis B Virus
DDLT	Deceased Donor Liver Transplantation
LT	Liver Transplantation
MASLD	Metabolic Dysfunction-Associated Steatotic Liver Disease
BMI	Body Mass Index
CCI	Comprehensive Complication Index
IQR	Interquartile Range
IRB	Institutional Review Board
RAC	Research Advisory Council
